# Autogenic succession and deterministic recovery following disturbance in soil bacterial communities

**DOI:** 10.1038/srep45691

**Published:** 2017-04-06

**Authors:** Stephanie D. Jurburg, Inês Nunes, James C. Stegen, Xavier Le Roux, Anders Priemé, Søren J. Sørensen, Joana Falcão Salles

**Affiliations:** 1 Genomic Research in Ecology and Evolution in Nature (GREEN), Groningen Institute for Evolutionary Life Sciences (GELIFES), University of Groningen, Nijenborgh 7, Groningen, 9747 AG, The Netherlands; 2 Section of Microbiology, University of Copenhagen, Universitetsparken 15, Building 1, 2100 Copenhagen, Denmark; 3 Earth and Biological Sciences, Biological Sciences Division, Pacific Northwest National Laboratory, Richland, WA, USA; 4 Microbial Ecology Center, INRA (UMR 1418), CNRS, Université Lyon1, Université de Lyon, 69622 Villeurbanne, France.

## Abstract

The response of bacterial communities to environmental change may affect local to global nutrient cycles. However the dynamics of these communities following disturbance are poorly understood, given that they are often evaluated over macro-ecological time scales and end-point measurements. In order to understand the successional trajectory of soil bacterial communities following disturbances and the mechanisms controlling these dynamics at a scale relevant for these organisms, we subjected soil microcosms to a heat disturbance and followed the community composition of active bacteria over 50 days. The disturbance imposed a strong selective pressure that persisted for up to 10 days, after which the importance of stochastic processes increased. Three successional stages were detected: a primary response in which surviving taxa increased in abundance; a secondary response phase during which community dynamics slowed down, and a stability phase (after 29 days), during which the community tended towards its original composition. Phylogenetic turnover patterns indicated that the community experienced stronger deterministic selection during recovery. Thus, soil bacterial communities, despite their extreme diversity and functional redundancy, respond to disturbances like many macro-ecological systems and exhibit path-dependent, autogenic dynamics during secondary succession. These results highlight the role of autogenic factors and successional dynamics in microbial recovery.

## Introduction

Soil microbes are essential components of ecosystems due to their contribution to global nutrient cycles. Disturbance effect is of central concern due to its potential to alter the functional capabilities of soil, and has been the subject of much research^1^,^2^. Previous studies have revealed that bacterial community recovery follows many of the general patterns observed in macro ecology, such as positive diversity-resilience and diversity-resistance relationships^3^,^4^,^5^. However a tendency to focus on single, end-point measurements of the recovering community has hampered a comprehensive understanding of the post-disturbance dynamics of bacterial communities during recovery, including the mechanisms controlling these dynamics. Importantly, the role that biotic interactions–or autogenic factors^6^ –may play in driving these dynamics has remained largely unexplored for microorganisms.

Microbial disturbance-recovery studies have generally relied on the assumption that the community will tend towards its pre-disturbance state, and that resistance is measured as the immediate changes to the community resulting from the disturbance, while resilience is measured as the long-term ability of the system to return to its pre-disturbance state^7^. However, a recent meta-analysis showed that microbial communities often do not recover to pre-disturbance levels within the wide range of experiments considered^8^. In this case, it is impossible to determine whether the system is still recovering or has recovered to an alternative stable state. Disturbances may exert constant, long-term abiotic pressures on the community, or they may be transient (pulse disturbance^8^). While a transient disturbance can induce major changes in abiotic pressures —affecting microbial communities in the short term— it can also induce longer-term cascading effects driven by biotic interactions and/or stochastic processes. In particular, previous studies have suggested that microbial community recovery is largely dependent on the community’s history, or legacy^9^,^10^,^11^,^12^,^13^. Therefore, in addition to characterizing the degree of return to an undisturbed state, it is equally important to understand the temporal dynamics of microbial communities following disturbance.

Moreover, mechanisms underlying the dynamics of secondary succession have extensively been studied for macro-ecological systems but are still poorly understood in bacterial communities^12^,^14^. Although there is still debate about how disturbance frequency and intensity influence successional processes, ecological theory regarding secondary succession generally posits that following disturbance, sequences of organisms colonize niche space made available by mortality of disturbance-sensitive taxa, leading eventually to a community that may differ substantially from both the pre-disturbance and initial post-disturbance communities^15^. Autogenic succession is largely mediated by direct or indirect competition for niche space, coupled with changes in the environment resulting from the colonization, as well as stochastic factors such as ecological drift and dispersal^16^. Autogenic secondary succession has been extensively explored in macro-ecology, particularly for primary producer communities^15^. For example, in the abandonment of an agricultural field, competition for light triggered by disturbance led to the favoring of tall-growing vegetation and the local extinction of the initial short-growing vegetation^17^.

Recent studies suggest that bacterial dynamics are deterministic and largely driven by individuals’ traits^18^. For example, a soil bacterial community inoculated into media at five different nutrient concentrations (representing nutrient availability along a successional gradient) showed that distinct and predictable communities are formed, depending on both nutrient concentration and time^19^. Similarly, in a study of bacterial recovery dynamics after rehydration of dry soil, a sequential increase of different groups of bacteria was observed, but whether the successional dynamics themselves played a role in the resulting sequence was unknown^20^. Taken together, these studies imply a degree of directionality in bacterial community succession, but the relative importance of stochasticity and determinism in driving post-disturbance dynamics of bacterial communities remain to be studied.

Here, we studied the recovery of soil bacterial communities in a controlled microcosm experiment in which soils were subjected to a transient disturbance in the form of a single heat shock and subsequently monitored over 50 days. Our primary aim was to examine the successional trajectory of bacterial communities following a disturbance, and evaluate the role of autogenic succession in driving changes in community composition. We hypothesized that following disturbance bacterial community dynamics would proceed in stages, initially determined by individuals’ tolerance to disturbance, and later determined by their ability to colonize open niche space, similar to the successional niche hypothesis in macro-ecology^21^. Thus, while disturbance would depress the abundance of sensitive organisms (which we term direct effects of the disturbance), the ensuing dynamics could result in a decrease in populations of resistant taxa and emergence of other taxa (indirect effects). An alternative hypothesis is that given extreme diversity and functional redundancy in bacterial systems, colonization may be phylogenetically stochastic^22^,^23^. This would indicate that the ability of different taxa to dominate a community at a particular point in time following disturbance may not be linked to their phylogenetic affiliation, due to a lack of relationship between phylogenetic affiliation and adaptation capacities. In this case, the niche space made available by the disturbance would be occupied by taxa independently of their affiliation, i.e. no community recovery patterns consistent from a taxonomic point of view would be observed following disturbance. Our results show that secondary succession in soil bacterial communities is similar to that of macro-ecological systems: it occurs in stages, and is largely prompted by biotic, or autogenic factors.

## Results

### Bacterial abundance and activity per cell

**Figure 1:**
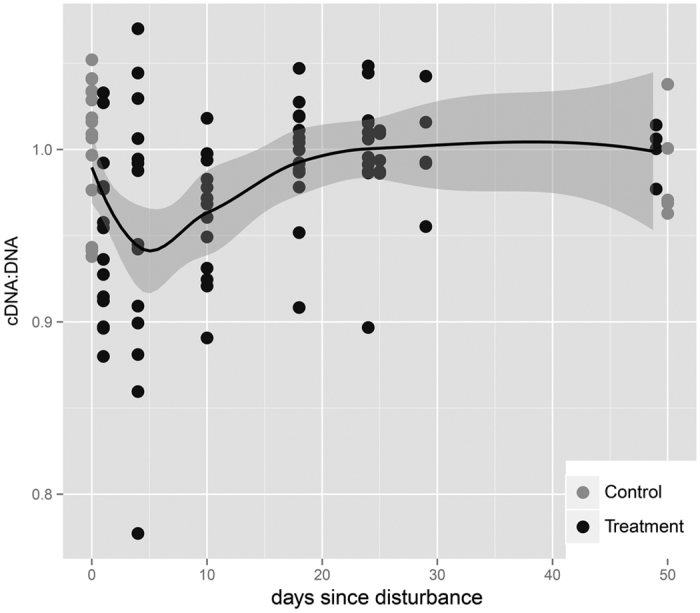
Bacterial activity rate during secondary succession. Average community activity is calculated as the ratio of cDNA:DNA 16S rRNA gene copy numbers. A lowess fit of the data is shown in black, with the standard error as grey shading. Separate cDNA and DNA measurements are available in Supplementary Information, S9.

The community-wide per-cell activity rate, calculated as the ratio of cDNA/DNA copies of the 16S rRNA gene, declined moderately following disturbance (one-tailed t-test, p = 0.054, 0.027, 0.057 for the comparison between values for pre-disturbance and T1, T4, and T10, respectively, Fig. 1), but reached pre-disturbance levels by T18 (one tailed t-test, p = 0.32) and remained indistinguishable from undisturbed soils thereafter (Supplementary Information, S1).

### Bacterial α-diversity

**Figure 2:**
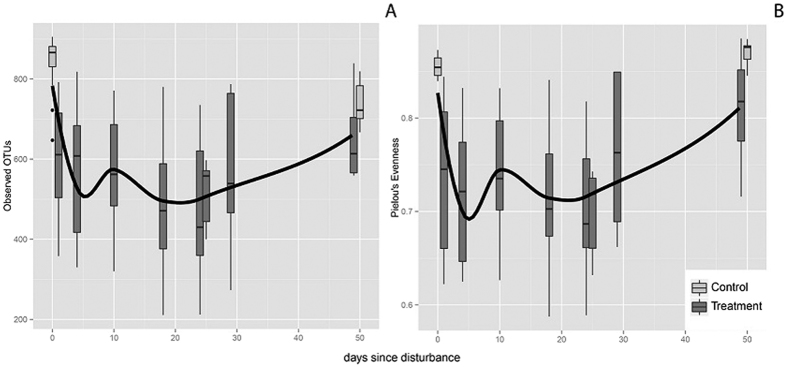
Variation in diversity during secondary succession. Richness (**A**) measured as the number of observed OTUs and evenness (**B**), calculated using Pielou’s J. A lowess fit of the data is shown in black.

Disturbance affected the potentially active bacterial community, with both richness (χ^2^ = 44.32, p = 0.012) and evenness (χ^2^ = 51.18 p < 0.001) decreasing after heating (Fig. 2). The mean OTU richness dropped by 28%, from 838 (±65.9 sd) in the undisturbed soils, to 602 (±148 sd) one day after disturbance. These differences persisted for over three weeks (χ^2^ post-hoc test; p < 0.005 for the comparison between T0 and T1-24), but subsequently began to recover. By T25 OTU richness was not significantly different from that of the C49. Evenness also decreased following disturbance (Fig. 2), with differences between disturbed communities and both T0 and C49 controls for up to 24 days post-disturbance (Tukey post-hoc test; p < 0.01 for all comparisons between T1-24 and T0, and T1-24 and C49). Evenness also fully recovered with respect to controls by T29.

### Bacterial β-diversity and community structure

**Figure 3:**
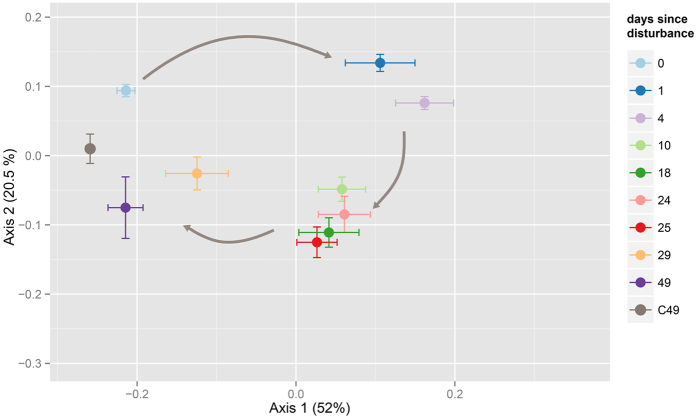
Variation in the community diversity during succession. A PCoA plot of weighted Unifrac distances between samples showed the strong effect of disturbance, and the temporally clustered pattern of recovery. Clusters were determined with a pairwise PERMANOVA p < 0.01. Centroids for each sampling time are shown along with their standard errors (error bars), and temporal dynamics are indicated with grey arrows.

The PCoA showed a significant effect of time since disturbance on community composition (PERMANOVA, pseudo F = 11.26, R^2^ = 0.54, p < 0.001, Fig. 3). Community composition of pre-disturbance (T0) soils and C49 controls were significantly different but clustered closely, and undisturbed community composition was relatively stable over the T0-T49 period as compared to disturbed communities (Supplementary Information, S2). Notably, disturbed communities temporally clustered into three groups according to the PERMANOVA, which we classified as response phases. The primary response phase, which encompassed T1-T4, involved a rapid shift along the first axis (52% of variation) away from pre-disturbance controls, as well as an increase in between-replicate variability (ANOVA of multivariate homogeneity of group dispersions, p < 0.001). This increased variability was also evident during the secondary response phase, T10-T25, which involved a shift along the second axis (20.5% of variation), away from both controls and communities characteristic of the primary response phase. The final stability phase (29–49 days after disturbance) marked the return of the communities towards the controls, predominantly along the first axis (Fig. 3). Samples from T49 clustered with C49 controls (pairwise PERMANOVA, p = 0.12). This last phase coincided with the return of richness and evenness to control levels. Bacterial communities associated with controls C1-C49 showed low variation, remaining similar throughout the experiment (Supplementary Information, S2).

### Bacterial succession

**Figure 4:**
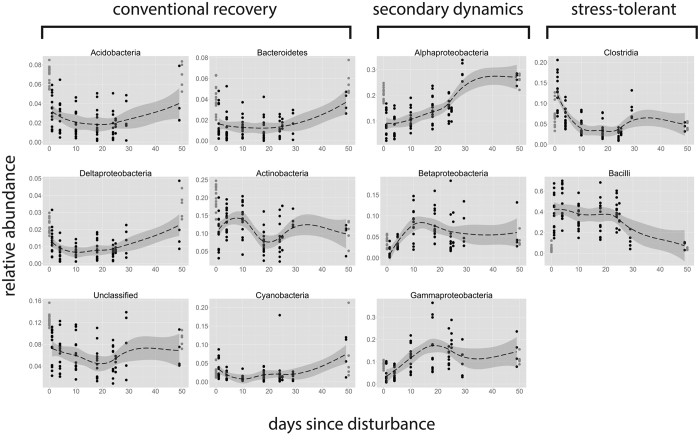
Phylum- and proteobacterial class-specific response patterns to disturbance. The responses of the relative abundance of dominant bacterial phyla, sorted according to the temporal patterns observed. From left to right, dominant phyla/classes exhibited either a *conventional recovery*, i.e. decrease following the disturbance and gradual recovery; *negative secondary dynamics*, i.e. negatively affected by the disturbance but rapidly recovering by the secondary response phase; or *survivors*, i.e. increase immediately after the disturbance, but gradual decrease thereafter. The groups displayed represent 96.5% of the community on average. The same data normalized by 16S rRNA abundance is available in Supplementary Information, S3.

Temporal responses were analyzed at the phylum, class and OTU levels in terms of the active community. At the phylum level, the pre-disturbance community was dominated by Proteobacteria (38 ± 5.5%), followed by Actinobacteria, Firmicutes and Acidobacteria (20.2 ± 6.3%, 12.1 ± 12.4% and 6.9 ± 1.5%, respectively), and 12.5 +/1.5% of the community was unclassified at the phylum level (Fig. 4). Dominant phyla exhibited temporal dynamics (Kruskal Wallis test, p < 0.01 for all comparisons), and were accordingly classified into three groups. A “conventional recovery” was observed for Acidobacteria, Deltaproteobacteria, Bacteroidetes, Actinobacteria, and unclassified OTUs, which were negatively affected by the disturbance (post-hoc Tukey test, p < 0.01 for comparison between T1 or T4 and T0), but exhibited a gradual recovery thereafter, approaching the relative abundances observed in control soils. Cyanobacterial relative abundances did not differ significantly over time (Fig. 4). “Positive secondary dynamics” were observed for Alphaproteobacteria, Betaproteobacteria, and Gammaproteobateria, which were negatively affected by the disturbance, but rapidly recovered after T4, reaching values even higher than the control values by T10 or T18 for Betaproteobacteria and Gammaproteobateria, and by T29 for Alphaproteobacteria (Fig. 4). “Stress tolerant” Clostridia and Bacilli exhibited a significant increase in relative abundance immediately after disturbance. The relative abundances of Clostridia then quickly decreased, while higher abundance of Bacilli persisted until day 25. In order to confirm that these relative abundance patterns reflected growth and death in Clostridia and Bacilli, and not growth/death in other bacterial taxa following disturbance, we normalized their abundances by the qPCR 16S rRNA counts, and found similar patterns (Supplementary Information, S3).

**Figure 5:**
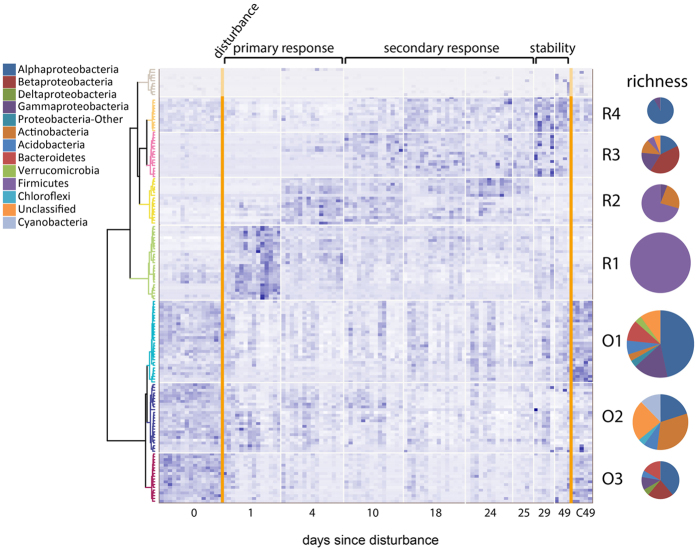
Temporal dynamics of bacterial OTU clusters along secondary succession. 156 OTUs which varied significantly in time were taken into account. Intensity of blue color indicates the relative abundance of each OTU. Phylum/subphylum membership (richness) within each group are shown in pie charts; pie chart size is scaled to the number of taxa represented in each group. Further details about each group are provided in Supplementary Information S4 and S5. The top cluster (grey) is composed of lowly abundant OTUs.

Two main response types were observed at the OTU level (Fig. 5). “Original” OTUs present in the undisturbed community were greatly suppressed by the disturbance, while the relative abundance of “Recovery” OTUs increased at some stage following the disturbance. These two main response types were further subdivided according to the temporal response patterns of the OTUs (Fig. 5). Within the Original groups, the relative abundances for OTUs in O1 decreased immediately following disturbance, while O2 and O3 were dominated by slow-growing bacteria such as *Nitrosospira* and *Ktedonobacter* or bacteria with very specific nutritional requirements such as *Phenylobacterium*, and declined further during the secondary response phase. The relative abundances of OTUs from O1 and O3 remained depressed for the duration of the experiment relative to controls (Supplementary Information, S4 and S5).

In contrast, the relative abundance of recovery groups peaked at different time points. Groups which peaked later exhibited higher taxonomic diversity: R1, which peaked immediately after the disturbance and then gradually decreased, contained only members of Firmicutes, including two strains of anaerobic *Clostridium* and two members of Planococcaceae. Group R2, which peaked 4 days after the disturbance, was dominated by Bacilli but also included the nutritionally diverse *Arthrobacter* and rapid-growing Proteobacteria such as *Pseudomonas.* Group R3 was the most diverse of the Recovery groups and included the slow-growing *Conexibacter*^24^ and another strain of *Phenylobacterium*. The relative abundance of OTUs in this group increased by day 10 and maintained this high abundance throughout the rest of the experiment, reaching an abundance which was two orders of magnitude greater than in the control by the end of the experiment. Group R4, which was dominated by nitrogen-fixing Proteobacteria (i.e. *Rhizomicrobium, Devosia,* and *Pseudolabrys*), was negatively affected by disturbance, but surpassed its pre-disturbance abundance by day 10, and remained higher than in both pre-disturbance and C49 controls.

### Stochastic vs. deterministic turnover during bacterial community recovery

**Figure 6:**
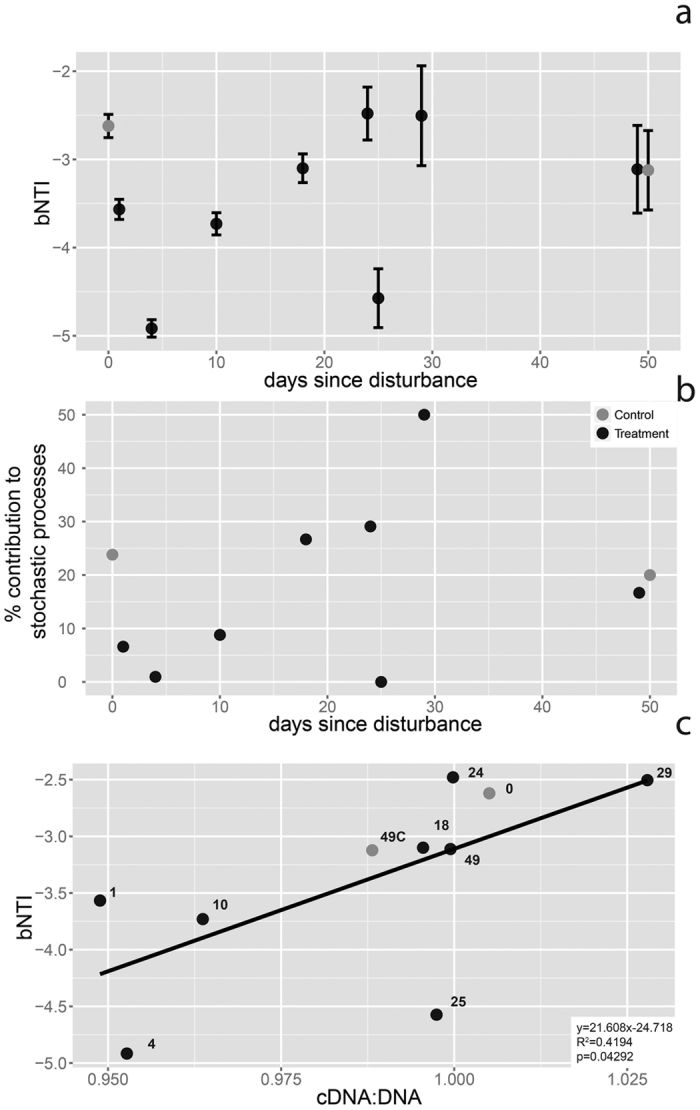
Type of phylogenetic turnover along recovery. (**a**) Temporal variation of the βNTI index. Values between 2 and −2 indicate stochastic turnover, while βNTI <−2 indicate homogeneous selection. (**b**) Temporal dynamics of the importance of stochasticity for the phylogenetic turnover, expressed as the percentage of comparison indicating stochasticity. (**c**) Relationship between βNTI and the 16S cDNA:rDNA ratio.

The undisturbed community exhibited a level of phylogenetic turnover consistent with mild homogeneous selection, whereby consistent selection pressures were imposed on the disturbed communities (βNTI mean = −2.62, Fig. 6A). The immediate effect of the disturbance was a significant increase in the strength of homogeneous selection (mean βNTI at T1 = −3.56, one-tailed t-test, p < 0.0001). This selective force was even stronger four days after disturbance (mean βNTI = −4.92) (one-tailed t-test between T1 and T4, p < 0.0001). The system gradually tended towards more stochastic turnover thereafter with highest values observed for T24 (βNTI mean = −2.47) and T29 (βNTI mean = −2.50), although an outlier value was obtained at T25. βNTI values became statistically indistinguishable from the undisturbed control at the end of the experiment.

Due to the variability of βNTI values, we also measured the proportion of comparisons for which βNTI indicated primarily stochastic turnover (|βNTI| < 2, Fig. 6B). The relative contribution of stochastic vs. deterministic processes first strongly decreased from 24% in the undisturbed community to 0% at T4. It then gradually increased until T29 and decreased to control levels by the T49 (Fig. 6B).

To further investigate whether the balance between stochastic versus deterministic turnover was related to average per-cell activity, we plotted average βNTI values against the ratio of cDNA:DNA counts (Fig. 6). A significant, positive relationship was observed between βNTI and average per-cell activity (cDNA:DNA, Fig. 6C).

## Discussion

Accounting for the role of autogenic factors and successional dynamics in microbial resilience is a critical step towards updating the current microbial disturbance-response framework. Community-wide measurements such as total potentially active bacteria and α-diversity are commonly used to evaluate the recovery of bacterial communities^1^,^8^. In terms of these measurements, our community followed “conventional” recovery patterns, similar to those observed in the literature for a variety of transient disturbances, in which rapid decreases in bacterial activity, community richness and evenness were found, followed by gradual returns to pre-disturbance conditions^1^,^2^. This resulted in partial or complete convergence with pre-disturbance parameters by the end of our study.

The return to pre-disturbance levels of community parameter values indicates that the system is resilient to the experimental heat shock within the period studied. However whether the underlying community is still reorganizing or whether the disturbance results in permanent changes to the recovered community is rarely examined^25^, and the mechanisms underlying these community dynamics are largely unknown. We found that the bacterial community structure recovered in temporally-clustered phases. These phases are consistent with the increase in the relative abundance of the temporal response groups outlined in Fig. 5. These response groups were phylogenetically congruent and, particularly for those which became more active during recovery, their composition was more variable in time.

The primary response stage, lasting up to four days after disturbance, coincided with a surge in the relative abundance of (generally heat-resistant) Firmicutes^26^. However, within this group there was some variation in the response: while Bacilli persisted until the stability phase, Clostridia rapidly decreased. This suggests that heat tolerance initially enabled survival, probably of spores, during the disturbance, which was followed by outgrowth and dominance of vegetative cells during the primary response phase. By the stability phase, the Firmicutes decreased to their pre-disturbance relative abundances, and taxa which had become depressed by the disturbance increased. At this stage, it is likely that other ecological properties, such as the ability to grow on recalcitrant carbon sources would have become more relevant for growth and competition outcome. The trajectory of Bacilli after the disturbance is analogous to the “survivor advantage” of trees with fire-resistant seeds, which are able to germinate before all other rapid colonizers arrive to burnt patches, and thus are able to dominate the patch at least for some time after a forest fire^27^.

The shift in abundance from the dominance of spore-forming (heat resistant) taxa to copiotrophic Proteobacteria and finally towards OTUs that were also present in the undisturbed communities suggests a gradual shift away from a disturbance-tolerant responder community. During the secondary response phase, copiotrophic taxa increased and slow-growing taxa which survived the heat disturbance decreased in relative abundance. We cannot determine the factors that may have triggered the shifts in composition from our experimental setup; however, the displacement of slow-growing taxa which were unaffected by the disturbance during the primary response phase and the importance of deterministic turnover following disturbance suggests that competition for resources between taxa intensified during the early stages of succession. For example, during the secondary response stage, we observed the reduction in the relative abundance of slow-growing taxa, such as *Nitrosospira* and *Ktedonobacter*^28^,^29^ which were active in the undisturbed community and survived the disturbance. Their decline coincided with the surge in rapidly-growing taxa, such as *Pseudomonas* and *Paeniporosarcina*. In particular, *Pseudomonas* is an opportunist, which often increases in abundance following disturbances^30^,^31^. A recent study found that nutrient concentration and type, as well as time since inoculation, were the main factors determining the structure of a soil community in a microcosm culture experiment, further supporting the notion that successional dynamics AMONG SOIL MICROBIOTA are often resource driven^19^. The role of resource competition has been repeatedly reported for successions in forests, where forests fires create open gaps, and competition for light drives sequential species replacements, and may even drive established fire-resistant species to extinction^15^. Our findings highlight the possibility of displacement resulting from resource competition during secondary succession. It is likely that feedbacks between surviving taxa and the environment (i.e., resource availability) gradually shifted the relative importance of different ecological traits during recovery: from survival, to rapid growth and labile resource use, and finally to recalcitrant resource use. Cell death caused by the disturbance would have released many resources into the soil, and this may have been a key driver of the dynamics observed during the secondary response phase (dominated by r-strategists) leading to stability (dominated by K-strategists). The gradual shift from r- to K-strategists along successional gradients has recently been shown for bacterial communities in simplified environments^18^. The relatively slow successional dynamics observed in our study may be due to the resource-poor (in terms of easily available nutrients) and fragmented conditions that are characteristic of aerated soils^32^. Furthermore, the role of other soil biota (in particular fungi, metazoans and archaea) - although outside the scope of this study - may have played, but may have played a role in modulating the successional phases observed and demands further study.

The sharp increase in deterministic turnover and homogeneous selection following the heat shock, which was stronger than in control samples for at least 10 days after disturbance suggests that a key, phylogenetically conserved trait (or traits) was very strongly selected for in the early phases of the post-disturbance landscape. The recovering community may have been subjected to a continuum of selective forces, i.e. first a strong abiotic filter imposed by heat shock, then the release from biotic competition due to lower densities of competing and/or antagonistic neighbors, and presumably increased resource availability, and finally the re-emergence of progressively stronger competition resulting from crowding and resource depletion. These results thus provide evidence for the possible influence of biotic interactions in shaping the community. However, correlated changes in taxa abundances are not necessarily the result of biotic responses but can also be due to indirect effects of modified available resources, which in turn might lead to changes in the community. Thus further work is needed to disentangle the importance of boitic interactions and resource availability in shaping microbial communities. The positive relationship between βNTI and average per-cell activity (cDNA:DNA) furthermore suggests that higher activity might reduce the importance of deterministic processes in shaping bacterial community assembly. In a previous study, it was suggested that deterministic factors may have a strong role after a soil disturbance and throughout the initial stages of secondary succession, without having the possibility to test this hypothesis and underlying mechanisms^33^. Due to the highly controlled setting in which our experiment was performed, we were able to explain the deterministic nature of the bacterial turnover following disturbance by biotic, or autogenic factors.

Our findings are consistent with a recent study which surveyed soil microbial communities in a range of field sites, and showed that traits related to survival (i.e. tolerance to desiccation and salt, formation of endospores and exospores) were favored under resource-limited conditions while traits related to competition for resources (i.e. phototrophic carbon fixation, denitrification, and formation of polyhydroxyalkanoate inclusions) were favored in high-resource conditions in microbial communities^34^. While it was previously established that individual traits scale up to bacterial community function^35^, our results indicate that individual traits are likely important drivers of community composition following disturbance, whereas stochastic processes are of less importance, providing novel mechanistic insight into bacterial community assembly during secondary succession.

Our findings shed light on bacterial resilience^1^ as a successional process. We provide strong evidence that biotic interactions drive the community dynamics for several days after disturbance and in direct response to disturbance, gradually steering the community away from the initial post- disturbance conformation. The existence of such directional dynamics suggests that the vulnerability of soil microbial communities to further perturbation is time-dependent, as well as dependent on the community’s disturbance legacy. Conversely, this may explain the success of strategies for managing microbial communities based on the application of several disturbances of increasing intensity^13^. Recent theoretical work suggests that resilience in the soil microbiota is dependent on the type and magnitude of the stresses experienced by the system in the past^12^. We further propose that because time since disturbance (i.e. successional stage) largely determines the community’s composition, it may play a crucial role in determining the system’s resilience: subjecting the system to a novel disturbance during the primary or secondary response phase is likely to result in a serious decrease in microbial diversity and in a potential collapse of the community, whereby internal feedbacks are permanently altered beyond repair^36^. Indeed, Kim and colleagues found that reinocculating soil microbial communities into sterile soils at different frequencies (every 7–56 days) led to increasingly deviant community compositions, and a collapse in the highest frequency^37^. Future work will be necessary to determine whether the temporal contingency of bacterial community recovery affects the community’s resilience to future perturbations.

## Materials and Methods

### Soil collection

Sandy loam soil (soil-water pH 5.04) was collected from the top 15 cm of a well-characterized agricultural field in Buinen, The Netherlands (52°55′N, 6°49′E) from four (2 × 2 m) plots in April 2013^38^,^39^. Following collection, soils were homogenized by sieving through a 4 mm sieve, moisture was adjusted to 65% water holding capacity (~58% in the field) with sterile water, and soils were allowed to stabilize for one month at 4 °C before filling the microcosms.

### Microcosms

A total of 120 microcosms were established by adding 50 g of fresh soil to 200 mL glass jars and covering them with a loose aluminum foil cap. Microcosms were maintained at 21 °C in a temperature and light-controlled greenhouse, and partially shielded from light by a single sheet of paper. Soil moisture was monitored and maintained weekly for the duration of the experiment. Soils were allowed to stabilize in the microcosms for two weeks prior to the beginning of the experiment. In order to study the dynamics of secondary succession in soil bacterial communities, it was necessary to apply a strong, quick and uniform disturbance, which was achieved by heat shocking the soil. The heat shock was administered by placing each uncovered jar in an 800 W microwave oven (R201ww Sharp, Utrecht, the Netherlands) for 90 s and heating to maximum intensity. Each jar was immediately adjusted for moisture loss and covered. The duration of the heat shock was selected after recording the effects of increasing durations of microwave heating (15 s to 10 min) on the total copies of 16S rRNA transcripts, soil temperature, pH, and moisture, in order to generate a loss of between 33% and 57% of 16S rRNA transcripts (data available in Supplementary Information, S6).

Fifteen microcosms were randomly chosen and harvested destructively at six time points: immediately before disturbance (T0), and at 1, 4, 10, 18, and 24 days after disturbance (T1-T24). In addition, five replicate microcosms were destructively harvested at 25, 29, 35, 42 and 49 days after disturbance (T25-49). Five non-treated, control microcosms were also sampled at the end of the experiment (C49), leading to a total of 120 microcosms. However, the 10 disturbed samples from T35 and T42, as well as seven other samples were excluded from further analyses due to problems linked to RNA extraction and low read numbers, resulting in 103 samples (see Supplementary Information, S7). To monitor the stability of undisturbed microcosms, we sampled 5 additional, control microcosms for each time point presented (T1-49) and measured their community composition and relative activity (available in Supplementary Information, S1 and S2).

### DNA and RNA extraction

DNA was extracted from 0.5 g of fresh, mixed soil using the MoBio PowerSoil DNA Extraction Kit (MoBio Laboratories, Carlsbad, CA, U.S.A.) following the manufacturer’s instructions, with three additional rounds of bead-beating for 30 s (mini-bead beater, BioSpec Products, Bartlesville, OK, U.S.A). Extracted products were run on a 0.8% agarose gel with a SmartLadder (Eurogentec, Liege, Belgium) to estimate the concentration and band-size for each sample.

For RNA extraction, 2 g of soil per sample were incubated for 24 hours at 4 °C in 5 ml of LifeGuard Soil Preservation Solution (MoBio Laboratories, Carlsbad, CA, USA), and kept in dry ice until extraction 7 days later. Extraction was performed using the RNA PowerSoil Total RNA Isolation Kit (MoBio Laboratories, Carlsbad, CA, USA) according to the manufacturer’s instructions. Extracts were re-suspended in 1 mM sodium citrate, quantified using a Quant-iT™ RNA AssayKit (range 5–100 ng; Invitrogen, Molecular approaches, OR, USA) on a Qubit^®^ fluorometer (Invitrogen, by Life Technologies, Nærum Denmark) and frozen at −80 °C. Ten samples which contained total RNA concentrations <20 ng μL^−1^ were discarded (Supplementary Information, S2). DNA was removed from each sample using the DNA-free™ Kit (Ambion^®^, by Life Technologies™, Nærum, Denmark) following manufacturer’s instructions and RNA was subsequently converted to cDNA using the Roche reverse transcription kit (Roche, Hvidovre, Denmark) with Random Hexameres (100 μM; TAG, Copenhagen, Denmark). Protocols are detailed in Supplementary Information S8.

### 16S rRNA gene copy number and transcript quantification

The numbers of 16S rRNA gene copies in the DNA and cDNA of each sample were used to estimate the numbers of bacterial cells and transcripts in the recovering communities. Copy number quantification targeting the 264-bp V5-V6 region of the 16S rRNA gene (primers 16SFP/16SRP^40^ was performed on an ABI PRISM 7300 Cycler (Applied Biosystems, Foster City, CA, U.S.A) as previously described^41^.

Each 25 μL reaction mixture contained 12.5 μL SYBR Green PCR Master Mix (Applied Biosystems, Foster City, CA, U.S.A.), 0.5 μL 20 mg mL^−1^ bovine serum albumin (Roche Diagnostics GmbH, Mannheim, Germany), 2 μL each of forward and reverse primers (10 mM), and 1 μL template DNA/cDNA at a concentration of 10 ng μL^−1^. Cycling conditions were as follows: 95 °C for 10 min, followed by 39 cycles of denaturation at 95 °C for 20 s, annealing at 62 °C for 60 s, and extension at 72 °C for 60 s. Fluorescence was detected after each annealing step. Product specificity was confirmed by melting curve analysis and checked on a 1.5% agarose gel. For each sample, gene copy number and transcript number were calculated from the DNA and cDNA respectively, using a standard curve spanning six orders of magnitude (10^2^–10^8^) as reference. The standard curve was generated using serial dilutions of plasmids containing a 16S rRNA gene cloned from a *Burkholderia sp.* For all runs, amplification efficiency ranged between 97.5–99%, and R^2^ of the serial dilutions was always greater than 97%. All data are shown as log gene copy number g^−1^ soil or log transcript number g^−1^ soil.

### 16S rRNA transcript sequencing and processing

cDNA obtained from 10 ng of total RNA was used for 16S rRNA gene transcript sequencing. A gene fragment of 460 bp flanking the V3 and V4 regions of the 16S rRNA gene was amplified using primers 341F and 806R^42^,^43^. Sequencing of the amplicons was performed using MiSeq reagent kit v2 (500 cycles) on a MiSeq sequencer (Illumina Inc., San Diego, CA, U.S.A). Additional information regarding sequencing conditions and processing methods is provided in Supplementary Information, S8. Briefly, paired end reads were merged and trimmed using Biopieces (www.biopieces.org) and UPARSE^44^. OTUs were clustered and their abundances were calculated using USEARCH^45^. OTUs were chimera-checked with UCHIME against the Greengenes 2011 database^46^. Singleton OTUs were removed. Following quality checking and trimming, we obtained 4 364 988 sequences with an average of 39 896 reads per sample (a range of 505 to 130 033 reads per sample).

### Statistical analyses

16S rRNA transcript sequences were used to evaluate the community composition of the active fraction of the bacterial community. Community analyses were carried out in R 3.2.3^47^ using the *vegan*^48^ and *Phyloseq*^49^ packages. Amplicon sequences were rarefied to an even depth of 3500 reads per sample for all downstream analyses. The rarefied dataset contained 3 826 OTUs, distributed over 103 samples.

The number of OTUs per sample was used as a measure of richness, and evenness was calculated using Pielou’s evenness index. To detect changes in α-diversity and the abundances of dominant phyla relative to the undisturbed soil, Kruskal-Wallis and post-hoc Tukey Nemeyi tests from package PMCMR^50^ were performed. In the case of ties, χ^2^ were used instead of Tukey. Time series were fitted with lowess curves in order to detect temporal trends. To evaluate changes in community composition over time, Principle Coordinates Analysis (PCoA) of weighted Unifrac distances was performed, and temporal patterns were quantified with a PERMANOVA using package *RVAideMemoire*^51^.

To identify successional groups (i.e. similarly responding taxa), we followed the procedure outlined in ref. 52: all OTUs which made up at least 0.5% of the community at least once throughout the experiment, appeared in three or more samples, and varied significantly with time since disturbance (ANOVA, p < 0.01) were selected. These 156 OTUs accounted for 70% (±22 sd) of the community on average throughout the experiment (data not shown). To confirm the validity of using this subset to represent the whole community, the full dataset and the subset of OTU’s were compared using procrustes analysis on Bray-Curtis dissimilarity matrices in the vegan package. This yielded a significant correlation of symmetric procrustes rotation (0.953, p = 0.001, 999 permutations), indicating that the two datasets were significantly similar. The abundance of each OTU was relativized over time to highlight changes in the temporal abundance patterns of each population. These temporal patterns were clustered by time (*vegan* package, euclidean distance, ward’s clustering). Inspection of the clustering pattern revealed eight groups of OTUs (confirmed using the *cutree* function of) with seven distinct temporal response patterns.

### Phylogenetic turnover quantification

In order to determine whether changes in community composition were more consistent with stochastic or deterministic turnover, we compared phylogenetic turnover to a null model. Phylogenetic turnover was quantified as the abundance weighted β-mean nearest taxon distance (βMNTD), and the results were compared to those expected under a completely stochastic system (null modeling approach). βMNTD was calculated using the R function *comdistnt* (abundance.weighted = TRUE; package *picante*). To quantify the magnitude and direction of deviation between an observed βMNTD value and the βMNTD value expected under stochastic community assembly, we used the β-nearest taxon index (βNTI), calculated as follows:


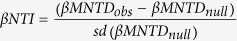


where βMNTD_obs_ is the observed βMNTD, βMNTD_null_ are null values of βMNTD, and sd indicates the standard deviation of the βMNTD_null_ distribution. We quantified βNTI for all pairwise comparisons, using a separate null model for each comparison. The distribution of βMNTD_null_ values was determined using 999 randomizations. In each randomization, OTUs were first randomly distributed across the tips of the phylogeny and βMNTD was subsequently quantified.

Statistically significant deviations from stochastic turnover have |βNTI| > 2. βNTI < −2 indicates homogeneous selection (sensu^33^), whereby the local environment imposes strong and consistent selection pressures, resulting in similar community composition across communities – i.e. indicating directional changes in community composition driven predominantly by a single factor. βNTI >+2 indicates variable selection (sensu ref. 33), whereby different communities are governed by different selection pressures (see also ref. 53). We considered all possible comparisons for βNTI within time points in order to evaluate whether the nature of phylogenetic turnover changed over time.

## Additional Information

**How to cite this article:** Jurburg, S. D. *et al*. Autogenic succession and deterministic recovery following disturbance in soil bacterial communities. *Sci. Rep.* **7**, 45691; doi: 10.1038/srep45691 (2017).

**Publisher's note:** Springer Nature remains neutral with regard to jurisdictional claims in published maps and institutional affiliations.

## Supplementary Material

Supplementary Information
