# Supplementary material for: Autogenic succession and deterministic recovery following disturbance in soil bacterial communities
Source: Sci Rep. 2017 Apr 6;7:45691. doi: 10.1038/srep45691 (PMC5382530; doi:10.1038/srep45691)
Supplement: Supplementary Information [file srep45691-s1.doc]

**Autogenic succession and deterministic recovery following disturbance in soil bacterial communities**

Jurburg SD, Nunes I, Stegen JC, Le Roux X, Priemé A, Sørensen SJ & Salles JF

**SUPPLEMENTARY INFORMATION**

**
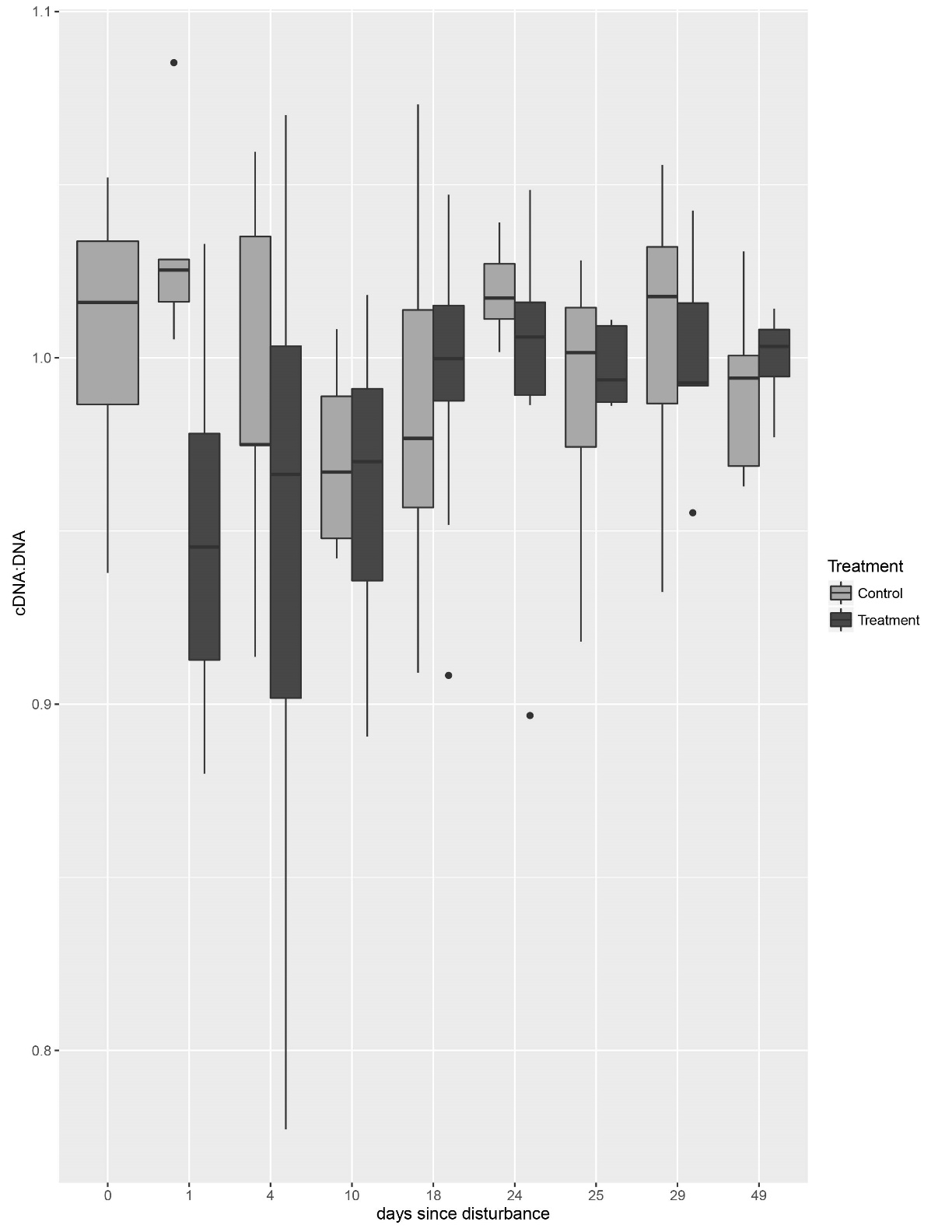
**

**S1. Bacterial activity rate during secondary succession including controls for each time point**. Community activity is calculated as the ratio of cDNA:DNA 16S gene copy numbers.

**
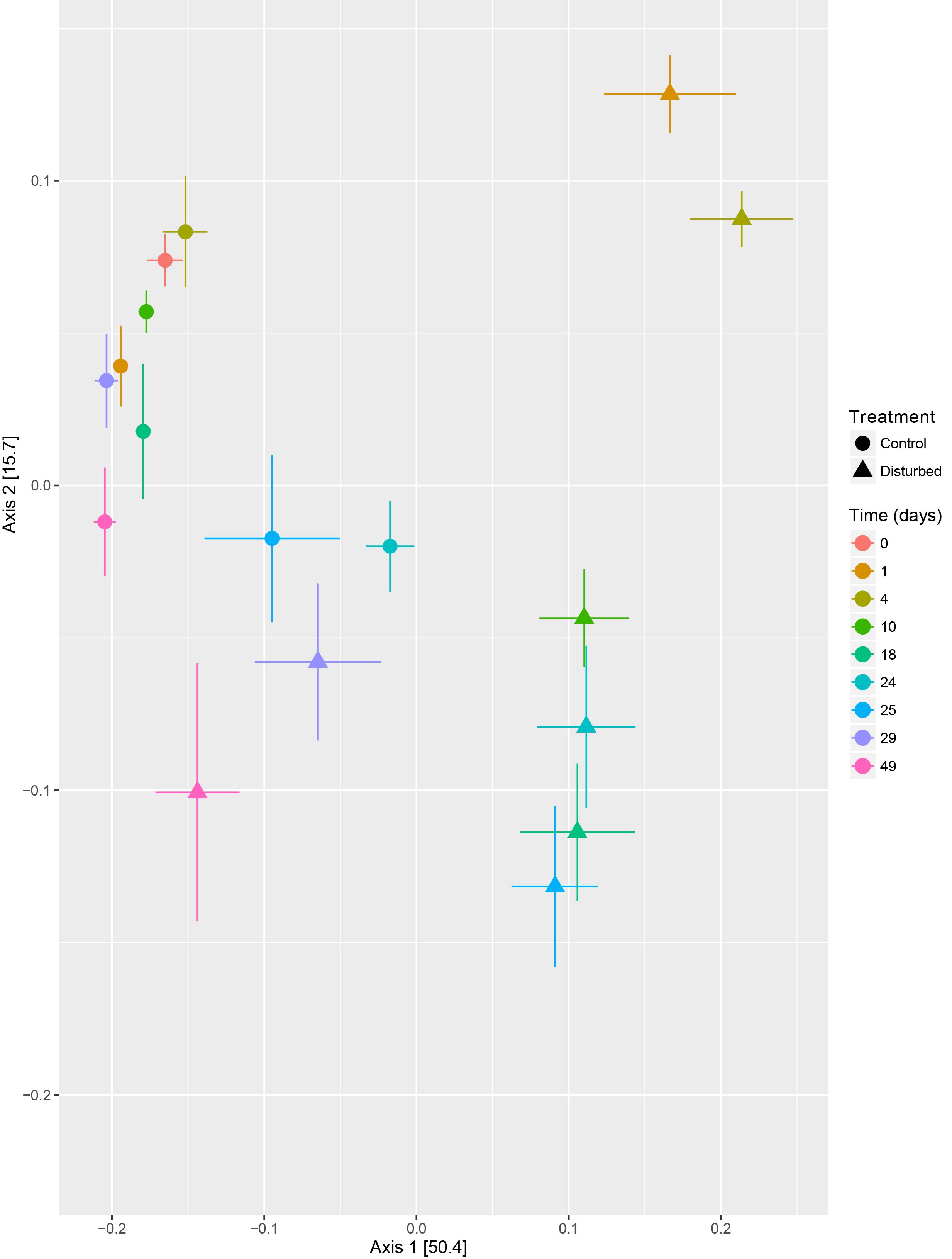
 S2. Variation of disturbed samples relative to control samples.** A PCoA plot of weighted Unifrac distances between disturbed and control samples over time. Centroids for each sampling time are shown along with their standard errors (error bars). The clustering of control samples indicates temporal stability relative to communities recovering from the disturbance.

**
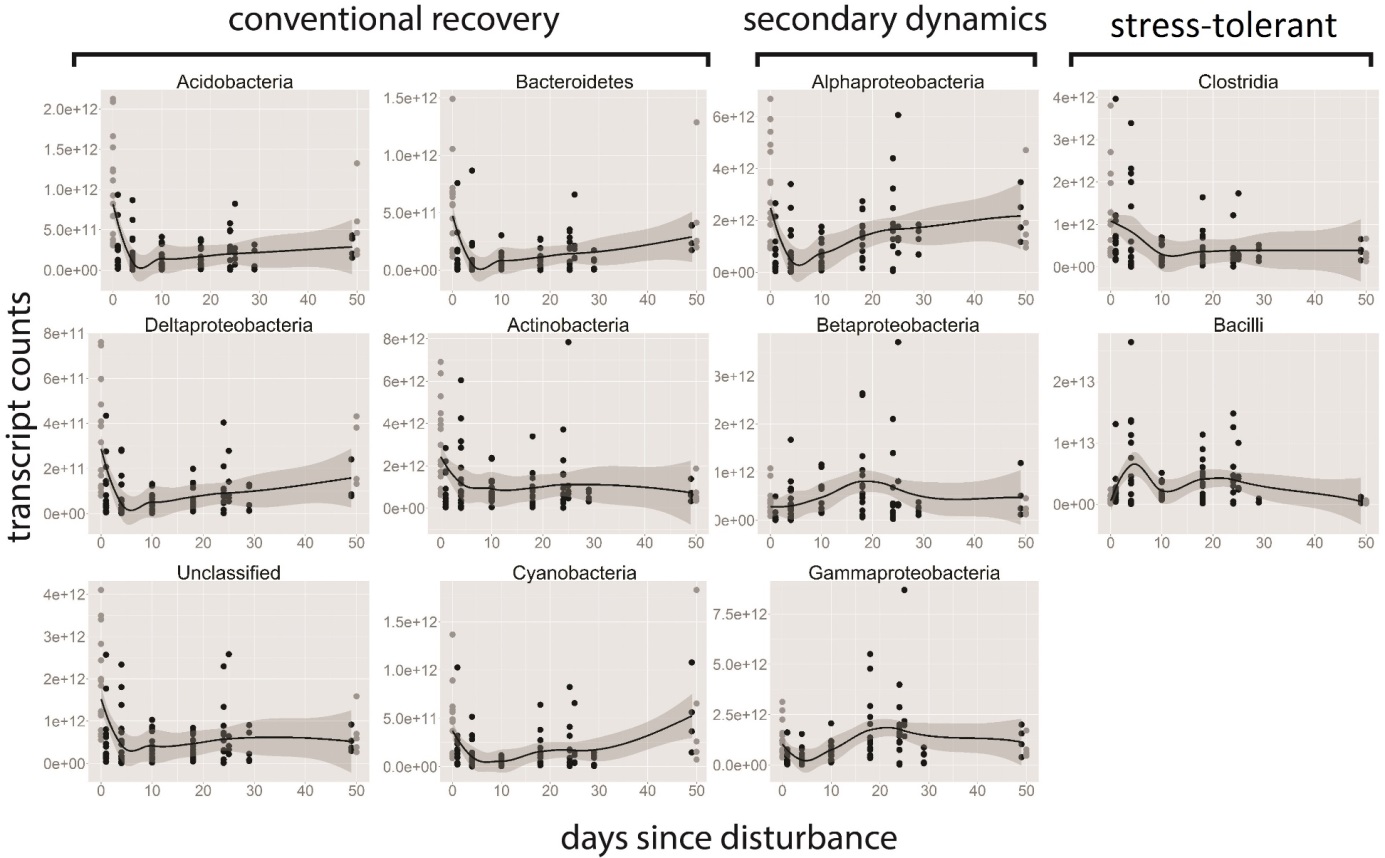
**

**S3. Phylum-specific response patterns to disturbance normalized by 16S rRNA copy counts**. The responses of the relative abundance of dominant bacterial phyla, sorted according to the temporal patterns observed. From left to right, dominant phyla /classes exhibited either a *conventional recovery*, i.e. decrease following the disturbance and gradual recovery; *negative secondary dynamics*, i.e. negatively affected by the disturbance but rapidly recovering by the secondary response phase; or *survivors*, i.e. increase immediately after the disturbance, but gradual decrease thereafter.

**S4. Functional response groups**. Five most abundant OTUs identify to the family and genus levels within each response group, ranked by abundance (top). These 35 OTUs made up 32.7±7% and 53.8±29.1% of the total abundance of the community in pre-disturbance and recovery samples, respectively. * Where an OTU could not be identified to at least the family level, the deepest classification available is provided.

| Group | Group Richness  (total Phyla, total OTUs) | Phylum | Family, genus |
| --- | --- | --- | --- |
| O1 | *5,30* | *Proteobacteria* | Unidentified *Gammaproteobacteria** |
|  |  | *Proteobacteria* | *Bradyrhizobiaceae, Bradyrhizobium* |
|  |  | Unidentified | Unidentified *Bacteria ** |
|  |  | *Proteobacteria* | *Xanthomonadaceae, Dokdonella* |
|  |  | *Bacteroidetes* | Unidentified *Chitinophagaceae** |
| O2 | *6,25* | *Actinobacteria* | *Micrococcaceae, Arthrobacter* |
|  |  | *Cyanobacteria* | *Cyanobacteria Family I, Group I* |
|  |  | *Actinobacteria* | Unclassfied *Solirubrobacterales** |
|  |  | *Cyanobacteria* | *Cyanobacteria Family I, Group I* |
|  |  | *Chloroflexi* | *Ktedonobacteraceae, Ktedonobacter* |
| O3 | *3,18* | *Proteobacteria* | *Nitrosomonadaceae, Nitrosospira* |
|  |  | *Proteobacteria* | *Comamonadaceae, Rhodoferax* |
|  |  | *Proteobacteria* | *Caulobacteraceae, Phenylobacterium* |
|  |  | *Proteobacteria* | *Xanthomonadaceae, Lysobacter* |
|  |  | *Bacteroidetes* | Unclassfied *Chitinophagaceae** |
| R1 | *1,27* | *Firmicutes* | Unclassified *Planococcaceae** |
|  |  | *Firmicutes* | *Planococcaceae, Sporosarcina* |
|  |  | *Firmicutes* | *Clostrdiaceae 1, Clostridium sensu stricto* |
|  |  | *Firmicutes* | *Clostrdiaceae 1, Clostridium sensu stricto* |
|  |  | *Firmicutes* | Unclassified *Bacilliales** |
| R2 | *3,17* | *Firmicutes* | *Planococcaceae, Planococcaceae incertae sedis* |
|  |  | *Firmicutes* | *Planococcaceae, Paenisporosarcina* |
|  |  | *Firmicutes* | *Paenibacillaceae 1, Paenibacillus* |
|  |  | *Proteobacteria* | *Pseudomonadaceae, Pseudomonas* |
|  |  | *Firmicutes* | Unclassified *Bacilliales** |
| R3 | *4,10* | *Proteobacteria* | *Xanthomonadaceae, Rhodanobacter* |
|  |  | *Proteobacteria* | *Caulobacteraceae, Phenylobacterium* |
|  |  | *Proteobacteria* | Unclassfied *Alcaligenaceae** |
|  |  | *Actinobacteria* | *Conexibacteraceae, Conexibacter* |
|  |  | *Proteobacteria* | *Burkholderiaceae, Burkholderia* |
| R4 | *1,12* | *Proteobacteria* | *Alphaproteobacteria incertae sedis, Rhizomicrbium* |
|  |  | *Proteobacteria* | *Hyphomicrobiaceae, Devosia* |
|  |  | *Proteobacteria* | *Caulobacteraceae, Phenylobacterium* |
|  |  | *Proteobacteria* | *Xanthomonadaceae, Dyella* |
|  |  | *Proteobacteria* | *Xanthobacteraceae, Pseudolabrys* |
|  |  |  |  |


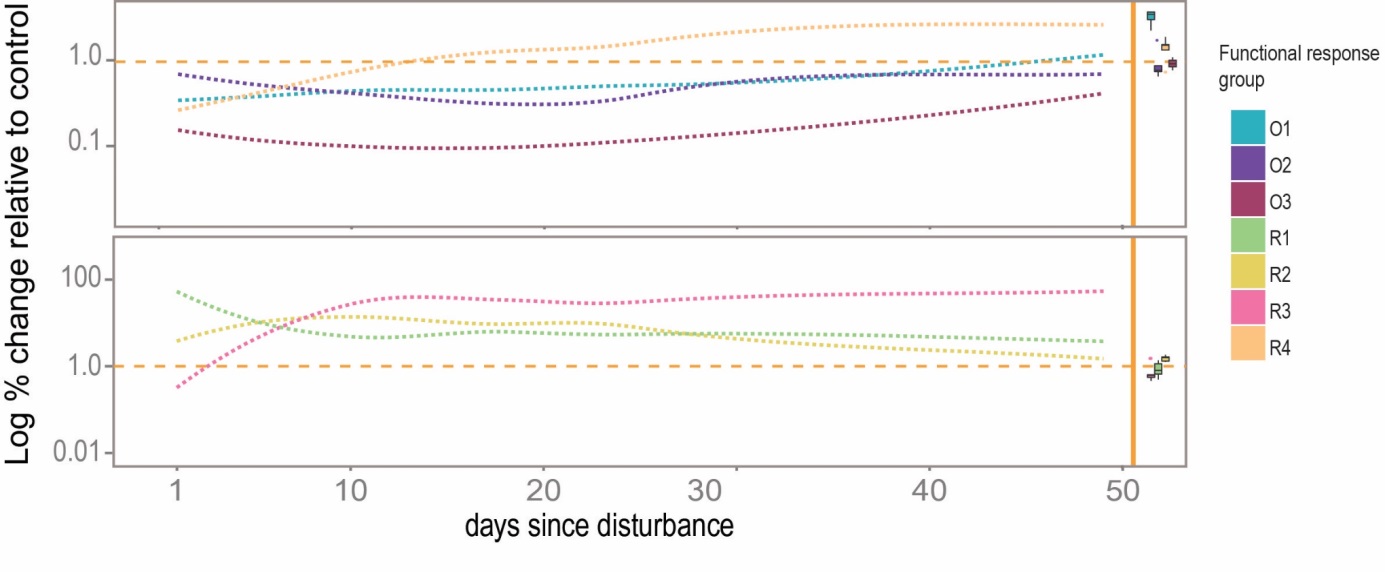


**S5. Functional response groups.** Loess-fitted curves show the average increase or decrease of each functional response group relative to the pre-disturbed communities (bottom). Box plots represent C49 controls

**S6. Effect of increasing exposure to microwave radiation on the soil microcosms.** No RNA was extractable at the highest exposures.

| **Duration** | **log gene copies of rRNA transcript p/gram dry soil** | **Temperature** | **pH** | **Water Loss**  **(g/g dry soil)** |
| --- | --- | --- | --- | --- |
| Control | 9.9 | 22 | 4.99 | 0.00 |
| Control | 10.7 | 21 | 4.97 | 0.00 |
| Control | 10.3 | 21 | 4.98 | 0.00 |
| Control | 10.5 | 27.5 | 4.95 | 0.01 |
| 15 s | 10.5 | 30 | 5 | 0.01 |
| 15 s | 9.72 | 27 | 4.98 | 0.01 |
| 15 s | 10.8 | 26 | 4.97 | 0.04 |
| 30 s | 10.2 | 39 | 4.99 | 0.02 |
| 30 s | 10.3 | 42 | 5 | 0.01 |
| 30 s | 10.2 | 34 | 4.5 | 0.01 |
| 30 s | 10.2 | 30 | 5.01 | 0.01 |
| 1 min | 10.2 | 55.5 | 4.97 | 0.02 |
| 1 min | 10.1 | 52 | 4.98 | 0.02 |
| 1 min | 10.1 | 54 | 5.01 | 0.01 |
| 1 min | 10.1 | 48 | 4.99 | 0.02 |
| 2 min | 9.84 | 72 | 4.95 | 0.02 |
| 2 min | 9.92 | 71 | 5 | 0.03 |
| 2 min | 9.86 | 55 | 4.96 | 0.03 |
| 2 min | 10.1 | 66 | 4.93 | 0.03 |
| 5 min | 9.28 | 72 | 4.95 | 0.12 |
| 5 min | 9.3 | 70 | 4.96 | 0.15 |
| 5 min | -- | 65 | 5.02 | 0.13 |
| 5 min | -- | 55 | 4.88 | 0.15 |
| 10 min | -- | 82 | 5.02 | 0.23 |
| 10 min | -- | 87 | 5.03 | 0.23 |
| 10 min | -- | 86 | 5.03 | 0.23 |
| 10 min | -- | 85.5 | 5.02 | 0.24 |

**S7. Summary of sample processing.** Cells in black indicate that the sample did not give data usable after RNA extraction. Cells in green indicate samples were removed due to low read numbers. Cells in orange indicate samples removed due RNA extraction problem for the other replicates from the same treatment. Control samples presented only in supplementary materials are italicized.

| | **Time since disturbance** | **Reads per sample** | **Sample name** | | --- | --- | --- | | 0 | 27501 | 3-D1 | | 0 | 33576 | 3-D2 | | 0 | 39654 | 3-D3 | | 0 | 41180 | 3-D4 | | 0 | 13961 | 3-D5 | | 0 | 3770 | 3-E1 | | 0 | 6425 | 3-E2 | | 0 | 43290 | 3-E3 | | 0 | 22051 | 3-E4 | | 0 | 35673 | 3-E5 | | 0 | 26523 | 3-F1 | | 0 | 40624 | 3-F2 | | 0 | 18814 | 3-F3 | | 0 | 31651 | 3-F4 | | 0 | 24936 | 3-F5 | | 1 | 30896 | 5-D1 | | 1 | 41696 | 5-D2 | | 1 | 68186 | 5-D3 | | 1 | 10719 | 5-D4 | | 1 | 25074 | 5-D5 | | 1 | 12336 | 5-E1 | | 1 | 48072 | 5-E2 | | 1 | 61467 | 5-E3 | | 1 | 52716 | 5-E4 | | 1 |  |  | | 1 | 46171 | 5-F1 | | 1 | 59780 | 5-F2 | | 1 | 57267 | 5-F3 | | 1 | 35215 | 5-F4 | | 1 | 65308 | 5-F5 | | 4 | 58490 | 6-D1 | | 4 | 45155 | 6-D2 | | 4 | 41526 | 6-D3 | | 4 | 61330 | 6-D4 | | 4 | 73563 | 6-D5 | | **Time since disturbance** | **Reads per sample** | **Sample name** | | 4 | 49283 | 6-E1 | | 4 | 46263 | 6-E2 | | 4 | 102182 | 6-E3 | | 4 | 26976 | 6-E4 | | 4 | 63334 | 6-E5 | | 4 | 36039 | 6-F1 | | 4 | 31011 | 6-F2 | | 4 | 69223 | 6-F3 | | 4 | 62410 | 6-F4 | | 4 | 50136 | 6-F5 | | 10 | 47264 | 7-D1 | | 10 | 25910 | 7-D2 | | 10 | 80412 | 7-D3 | | 10 | 130033 | 7-D4 | | 10 | 101304 | 7-D5 | | 10 | 76745 | 7-E1 | | 10 | 57248 | 7-E2 | | 10 | 98197 | 7-E3 | | 10 | 46045 | 7-E4 | | 10 | 74947 | 7-E5 | | 10 | 19266 | 7-F1 | | 10 | 505 |  | | 10 | 60557 | 7-F3 | | 10 | 41510 | 7-F4 | | 10 | 53606 | 7-F5 | | 18 | 94806 | 8-D1 | | 18 | 34460 | 8-D2 | | 18 | 79785 | 8-D3 | | 18 | 36053 | 8-D4 | | 18 | 42980 | 8-D5 | | 18 | 73801 | 8-E1 | | 18 | 21005 | 8-E2 | | 18 | 9969 | 8-E3 | | 18 | 32370 | 8-E4 | | 18 | 16998 | 8-E5 | | 18 | 37006 | 8-F1 | | 18 | 6050 | 8-F2 | | 18 | 30151 | 8-F3 | | 18 | 35814 | 8-F4 | | 18 | 23073 | 8-F5 | | 24 | 26987 | 9-D1 | | 24 | 21973 | 9-D2 | | 24 | 45505 | 9-D3 | | 24 | 38587 | 9-D4 | | 24 | 28818 | 9-D5 | | 24 | 47831 | 9-E1 | | 24 | 47148 | 9-E2 | | 24 | 61143 | 9-E3 | | 24 | 36998 | 9-E4 | | 24 | 37163 | 9-E5 | | 24 |  |  | | 24 | 45354 | 9-F2 | | 24 |  |  | | 24 |  |  | | 24 |  |  | | 25 | 51417 | 11-E1 | | 25 | 69635 | 11-E2 | | 25 | 30070 | 11-E3 | | 25 | 24444 | 11-E4 | | 25 | 38388 | 11-E5 | | 29 | 21525 | 12-E1 | | 29 | 53062 | 12-E2 | | 29 | 24850 | 12-E3 | | 29 | 67371 | 12-E4 | | 29 | 93554 | 12-E5 | | 35 | 660 |  | | 35 | 789 |  | | 35 | 6035 |  | | 35 | 1479 |  | | 35 | 2421 |  | | 42 |  |  | | 42 | 21651 |  | | 42 |  |  | | 42 |  |  | | 42 |  |  | | 49 | 17409 | 15-E1 | | 49 | 22896 | 15-E2 | | 49 | 10204 | 15-E3 | | 49 |  |  | | 49 | 11128 | 15-E5 | | C49 | 23922 | 15-C1 | | C49 | 8198 | 15-C2 | | C49 | 5040 | 15-C3 | | C49 | 10073 | 15-C4 | | C49 | 5542 | 15-C5 | | *C1* | *22882* | *5-C1* | | *C1* | *26425* | *5-C2* | | *C1* | *26554* | *5-C3* | | *C1* | *49892* | *5-C4* | | *C1* | *20244* | *5-C5* | | *C4* | *32799* | *6-C1* | | *C4* | *66725* | *6-C2* | | *C4* | *19391* | *6-C3* | | *C4* | *76320* | *6-C4* | | *C4* | *60753* | *6-C5* | | *C10* | *58643* | *7-C1* | | *C10* | *50699* | *7-C2* | | *C10* | *38236* | *7-C3* | | *C10* | *49570* | *7-C4* | | *C10* | *51971* | *7-C5* | | *C18* | *46172* | *8-C1* | | *C18* | *17882* | *8-C2* | | *C18* | *24087* | *8-C3* | | *C18* | *17666* | *8-C4* | | *C18* | *25711* | *8-C5* | | *C24* | *34551* | *9-C1* | | *C24* | *63482* | *9-C2* | | *C24* | *27551* | *9-C3* | | *C24* | *15197* | *9-C4* | | *C24* | *51513* | *9-C5* | | *C25* | *28284* | *11-C1* | | *C25* | *4576* | *11-C2* | | *C25* | *74433* | *11-C3* | | *C25* |  | *11-C4* | | *C25* | *27910* | *11-C5* | | *C29* | *26533* | *12-C1* | | *C29* | *58916* | *12-C2* | | *C29* | *20034* | *12-C3* | | *C29* | *52112* | *12-C4* | | *C29* | *49814* | *12-C5* | | *C35* | *2745* |  | | *C35* | *705* |  | | *C35* | *33739* | *13-C3* | | *C35* | *21778* | *13-C4* | | *C35* | *15099* | *13-C5* | | *C42* | *9530* | *14-C1* | | *C42* | *25979* | *14-C2* | | *C42* | *20087* | *14-C3* | | *C42* | *1230* |  | | *C42* | *21847* | *14-C5* | |  |  |  |
| --- | --- | --- | --- | --- | --- | --- | --- | --- | --- | --- | --- | --- | --- | --- | --- | --- | --- | --- | --- | --- | --- | --- | --- | --- | --- | --- | --- | --- | --- | --- | --- | --- | --- | --- | --- | --- | --- | --- | --- | --- | --- | --- | --- | --- | --- | --- | --- | --- | --- | --- | --- | --- | --- | --- | --- | --- | --- | --- | --- | --- | --- | --- | --- | --- | --- | --- | --- | --- | --- | --- | --- | --- | --- | --- | --- | --- | --- | --- | --- | --- | --- | --- | --- | --- | --- | --- | --- | --- | --- | --- | --- | --- | --- | --- | --- | --- | --- | --- | --- | --- | --- | --- | --- | --- | --- | --- | --- | --- | --- | --- | --- | --- | --- | --- | --- | --- | --- | --- | --- | --- | --- | --- | --- | --- | --- | --- | --- | --- | --- | --- | --- | --- | --- | --- | --- | --- | --- | --- | --- | --- | --- | --- | --- | --- | --- | --- | --- | --- | --- | --- | --- | --- | --- | --- | --- | --- | --- | --- | --- | --- | --- | --- | --- | --- | --- | --- | --- | --- | --- | --- | --- | --- | --- | --- | --- | --- | --- | --- | --- | --- | --- | --- | --- | --- | --- | --- | --- | --- | --- | --- | --- | --- | --- | --- | --- | --- | --- | --- | --- | --- | --- | --- | --- | --- | --- | --- | --- | --- | --- | --- | --- | --- | --- | --- | --- | --- | --- | --- | --- | --- | --- | --- | --- | --- | --- | --- | --- | --- | --- | --- | --- | --- | --- | --- | --- | --- | --- | --- | --- | --- | --- | --- | --- | --- | --- | --- | --- | --- | --- | --- | --- | --- | --- | --- | --- | --- | --- | --- | --- | --- | --- | --- | --- | --- | --- | --- | --- | --- | --- | --- | --- | --- | --- | --- | --- | --- | --- | --- | --- | --- | --- | --- | --- | --- | --- | --- | --- | --- | --- | --- | --- | --- | --- | --- | --- | --- | --- | --- | --- | --- | --- | --- | --- | --- | --- | --- | --- | --- | --- | --- | --- | --- | --- | --- | --- | --- | --- | --- | --- | --- | --- | --- | --- | --- | --- | --- | --- | --- | --- | --- | --- | --- | --- | --- | --- | --- | --- | --- | --- | --- | --- | --- | --- | --- | --- | --- | --- | --- | --- | --- | --- | --- | --- | --- | --- | --- | --- | --- | --- | --- | --- | --- | --- | --- | --- | --- | --- | --- | --- | --- | --- | --- | --- | --- | --- | --- | --- | --- | --- | --- | --- | --- | --- | --- | --- | --- | --- | --- | --- | --- | --- | --- | --- | --- | --- | --- | --- | --- | --- | --- | --- | --- | --- | --- | --- | --- | --- | --- | --- | --- | --- | --- | --- | --- | --- | --- | --- | --- | --- | --- | --- | --- | --- | --- | --- | --- | --- | --- | --- | --- | --- | --- | --- | --- | --- | --- | --- | --- | --- | --- | --- | --- | --- | --- | --- | --- | --- | --- | --- | --- | --- | --- | --- | --- | --- | --- | --- | --- | --- | --- | --- | --- | --- | --- | --- | --- | --- | --- | --- | --- | --- | --- | --- | --- | --- | --- | --- | --- | --- | --- | --- | --- | --- | --- | --- | --- | --- | --- | --- | --- | --- | --- | --- | --- | --- | --- | --- | --- | --- | --- | --- | --- | --- | --- |

**S8. Supplementary materials and methods**

**RNA processing**

200 ng of total RNA were treated using 2 µL of 10X Dnase I buffer and 1 µL of DNase I. Incubation was performed for 1h at 37°C with mixing at each 10 minutes. 5 µL of DNase inactivation reagent were then added and incubation at room temperature for 5 minutes with frequent mixing for precipitate re-suspension was made. Supernatant was recovered after centrifugation at 10000 x g cDNA was obtained using the Roche reverse transcription kit (Roche, Hvidovre, Denmark) with Random Hexameres (100 µM; TAG Copenhagen, Denmark). Reaction mixes were constituted by 4 µL of 5x Strand buffer, 2 µL of DTT (0.1 M), 1 µL of dNTP (10 mM), 1 µL of Random Hexameres, 2 µL of template DNase treated RNA and water to a total volume of 20 µL. Mixes were incubated in a Peltier Thermal Cycler (DNA Engine DYAD™, USA) in a one cycle protocol as following: 2 minutes at 42°C followed by the addition of 1 µL of reverse transcriptase (50 U/µL; Roche, Hvidovre, Denmark), 40 minutes at 42°C, 30 minutes at 50°C and 15 minutes at 72°C to stop the reaction.

**16S rRNA transcript sequencing**

The primers 341F (5’CCTACGGGRBGCASCAG-3’) and 806R (5’GGACTACNNGGGTATCTAAT-3’) (Sigma-Aldrich, Brøndby, Denmark) flanking the V3 and V4 regions of the 16S rRNA gene were used to amplify a gene fragment of 460 bp. The chosen primer set have previously been successfully used to target bacteria and archaea but with a higher matching efficiency for bacteria (Yu *at al.*, 2005; Berg *et al.*, 2012). The PCR mixes were constituted by 2.0 µL of 10X AccuPrime™ PCR Buffer I containing 15 mM of MgCl2 (Life Technologies, Nærum, Denmark), 0.12 µL AccuPrime™ Taq DNA Polymerase (2 units/µl, Life Technologies, Nærum, Denmark), 1 µL of each primer (10 µM), 1 µL of a 1:10 dilution of cDNA as template and water to a total volume of 20 µL. The first PCR reactions were performed in a Peltier Thermal Cycler (DNA Engine DYAD™, USA) according to the following conditions: an initial activation of the hotstart polymerase at 94°C for 2 min, followed by 35 cycles of denaturation at 94°C for 20 s, annealing at 56°C for 20s and extension at 68°C for 30s, with a final extension at 68°C for 5 min. Concentration of amplified PCR products was measured by Pico Green (Life Technologies, Nærum, Denmark) using a LightCycler 96 (Roche, Hvidovre, Denmark). Addition of adapters and indexes to DNA fragments was done in a second PCR where mixes were composed by: 2.0 µL of 10X AccuPrime™ PCR Buffer II containing 15 mM of MgCl2 (Invitrogen, Tåstrup, Denmark), 0.12 µL of AccuPrime™ Taq DNA Polymerase (2 units/µl, Life Technologies, Nærum, Denmark), 1.0 µL of each fusion primers (MS_515f_F IndexNo and MS_806r_R IndexNo; 10 µM), 2 µL of PCR product obtained in the previous PCR and water to a total of 20 µL. A Peltier Thermal Cycler (DNA Engine DYAD™, USA) was used to perform the second PCR reactions in the following way: initial activation of the hotstart polymerase at 94°C for 2 min, followed by 15 cycles of denaturation at 94°C for 20 s, annealing at 56°C for 20s and extension at 68°C for 30s, with final extension at 68°C for 5 min. The amplified PCR products were incubated at 70°C for 3 min and then placed on ice. Obtained fragment has approximately 400 bp including tag primers. Purification of PCR products was obtained using the AmPure XP Bead Purification Kit (Beckman Coulter, Copenhagen, Denmark) accordingly to the manufacturer instructions and applying 15 µL of AgencourtAMPure XP per reaction (0.75X of the total volume). The concentration of the purified second PCR products was measured by Pico Green (Life Technologies, Nærum, Denmark) in a LightCycler 96 (Roche, Hvidovre, Denmark) and equal amounts of DNA were pooled in a 1.5 mL tube. The pooled sample was concentrated using DNA clean and concentrator-5 kit (Zymo Research, Irvine, CA, USA).

**Sequence processing**

Paired end reads were merged and primer remnants were trimmed using Biopieces (www.biopieces.org). For merging, reads were aligned and reads with mismatches filtered out. Reads were quality filtered using the UPARSE algorithm (Edgar 2013) with a maximum expected error rate of 0.5. OTUs were clustered at 97% and abundances calculated by mapping reads back to OTUs using USEARCH(Edgar 2010). OTUs were chimera-checked with UCHIME against the Greengenes 2011 database (DeSanti*s et a*l. 2006), in-silico amplified with Biopieces to only contain the V4 region. Sequences were filtered for non-bacterial contaminants by clustering against the RDP trainset PDS (mitochondria added) v9 at 60%. Singleton OTUs were removed. Representative reads picked by USEARCH were classified using the Wang implementation of Mothur (Schlos*s et a*l. 2009) against the RDP trainset PDS v9. Classifications were accepted at a threshold of 80% confidence for each taxonomic level.

**
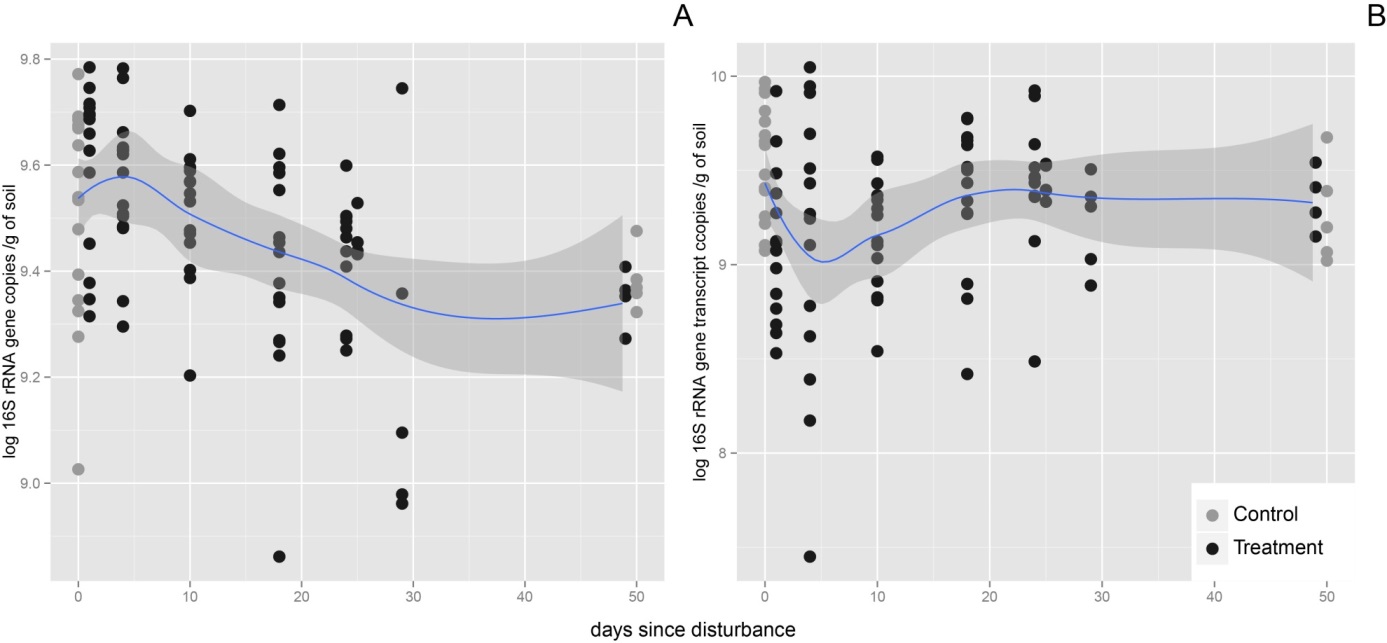
**

**S9. Total (A) and transcriptionally active (B) bacterial community following disturbance**.

A lowess fit of the data is shown in blue, with the standard error as grey shading.
